# Benefits of group compassion-focused therapy for treatment-resistant depression: A pilot randomized controlled trial

**DOI:** 10.3389/fpsyg.2022.903842

**Published:** 2022-08-12

**Authors:** Kenichi Asano, Masao Tsuchiya, Yoko Okamoto, Toshiyuki Ohtani, Toshihiko Sensui, Akihiro Masuyama, Ayako Isato, Masami Shoji, Tetsuya Shiraishi, Eiji Shimizu, Chris Irons, Paul Gilbert

**Affiliations:** ^1^Department of Psychological Counseling, Faculty of Psychology, Mejiro University, Tokyo, Japan; ^2^The Japanese Centre for Compassionate Mind Research and Training, Tokyo, Japan; ^3^Advantage Risk Management Co. Ltd., Meguro-ku, Japan; ^4^Research Center for Child Mental Development, Chiba University, Chiba, Japan; ^5^Safety and Health Organization, Chiba University, Chiba, Japan; ^6^Department of Psychology, Faculty of Humanities, Saitama Gakuen University, Saitama, Japan; ^7^Faculty of Psychology, Iryo Sosei University, Fukushima, Japan; ^8^Department of Psychiatry, Kokoronomori Clinic, Chiba, Japan; ^9^Balanced Minds, London, United Kingdom; ^10^Centre for Compassion Research and Training, College of Health, Psychology and Social Care, University of Derby, Derby, United Kingdom; ^11^The Compassionate Mind Foundation, Derby, United Kingdom

**Keywords:** compassion focused therapy (CFT), treatment-resistant depression, self-compassion, randomized controlled trial (RCT), compassion, group psychotherapy

## Abstract

Major depression is one of the most common mental health problems worldwide. More than one-third of patients suffer from treatment-resistant depression (TRD). In this study, we explored the feasibility of group compassion-focused therapy (CFT) for TRD using a randomized controlled trial with two parallel groups. Eighteen participants were randomly allocated to the intervention group (CFT and usual care) and control group (usual care alone) and a participant in each group withdrew. Participants in the intervention group received a 1.5-h session every week for 12 weeks. The effects of the intervention on the participants’ scores were calculated using a linear mixed model. There was a larger reduction in their depressive symptoms and fears of compassion for self and a greater increase in their compassion for self compared to the control group participants. The reliable clinical indices showed that in the CFT (intervention) group, three of nine participants recovered (33%), two improved (22%), two recovered but non-reliably (22%), and the condition of two remained unchanged (22%). These findings indicate adequate feasibility of group CFT for TRD in Japanese clinical settings.

**Clinical trial registration:**

[https://clinicaltrials.gov/], identifier [UMIN 000028698].

## Introduction

Major depression is a common mental health problem worldwide. James et al. (2018) reported that more than 264 million people suffered from depressive disorders. The situation is similar in Japan, where the lifetime prevalence of mood disorders is 4.12% in men and 9.80% in women, and the 12-month prevalence is 1.29% in men and 3.70% in women (Nishi et al., 2019).

Various guidelines recommend cognitive behavioral therapy (CBT) as a psychological treatment for major depression (National Institute for Health and Care Excellence, 2009; American Psychological Association, 2019). However, despite treatment, some patients struggle with chronic symptoms and experience treatment-resistant depression (TRD; EMEA, 2009). Although the definition of TRD is not universal, it is most commonly defined as “an inadequate response after at least 2 antidepressant trials of adequate dose, duration, and treatment adherence” (Thase, 2011). Rush et al. (2006) found that over 50% of patients diagnosed with depression do not remit after first-line antidepressant medication, and approximately 30% may not remit after multiple treatments. Thomas et al. (2013) reported that 55% of patients with major depressive disorder were eligible for a diagnosis of TRD. These findings highlight the importance of treating chronic depression. However, patients with TRD present complex clinical problems related to multiple risk factors and may need novel psychotherapies (Al-Harbi, 2012).

For treating chronic depression or TRD with psychological intervention, the National Collaborating Centre for Mental Health (2011) guidelines recommend Dialectical behavior therapy (DBT), CBT, Interpersonal Psychotherapy (IPT), and short-term dynamic psychotherapy. A systematic review assessing the effectiveness of psychological interventions reported that although these psychotherapies delivered to patients were reliable, further evidence is needed on the effectiveness of the different types of psychotherapy (Ijaz et al., 2018).

Relatedly, psychological interventions focused on compassion have been garnering attention in recent years. This trend is evidenced by the emergence of various concepts related to compassion. The most popular one is self-compassion, defined as “being open to and moved by one’s own suffering, experiencing feelings of caring and kindness toward oneself, taking an understanding, non-judgmental attitude toward one’s inadequacies and failures, and recognizing that one’s own experience is part of the common human experience” (Neff, 2003). Meta-analyses of cross-sectional data have revealed that self-compassion is negatively correlated with psychopathology (MacBeth and Gumley, 2012) and psychological distress (Marsh et al., 2018) and positively correlated with well-being (Zessin et al., 2015), physical health and health behavior (Liao et al., 2021; Phillips and Hine, 2021), physical activity (Wong et al., 2021), and sleep quality (Brown et al., 2021). These findings indicate that enhancing or developing self-compassion can promote mental and physical health. However, this concept and measure of self-compassion can be difficult to interpret because it combines positive factors such as kindness with “negative” factors of self-criticism which have long known been linked to depression and for which there many different therapies (Ferrari et al., 2022; Muris and Otgaar, 2022). In addition MSC was not (originally) developed as a psychotherapy but for self-help (Neff, 2003).

Meta-analyses of different types of compassion-based interventions (CBIs) with clinical applications have also been conducted. Wilson et al. (2019) showed that self-compassion-related therapies (compassion focused therapy, mindfulness-based cognitive therapy, and acceptance and commitment therapy) improved anxiety and depression, based on the data from 22 randomized controlled trials (RCTs). Kirby et al. (2017) also revealed that CBIs significantly improved scores related to mental health or well-being. Craig et al. (2020) systematic review with meta-analysis found that compassion focused therapy (CFT) reduced depression and anxiety and could be an alternative treatment for severe and complex mental health problems.

Indeed, two studies specifically investigated CBIs for chronic depression or TRD. Graser et al. (2016) pilot study with a single-group design examined the effectiveness of a 12-week mixed-group program on mindfulness, CFT, and loving kindness meditation. The results showed that the symptoms of patients with chronic depression decreased after participating in the program; this progress was also observed in the three-month follow-up. Moreover, in Asano and Shimizu (2018) case study, a patient with recurrent depression responded to individual CFT. These reports suggest the potential effectiveness of CBIs for TRD, which is also supported by evidence that CBIs reduce self-criticism and shame. Self-criticism has long been shown to be associated with depressive symptoms and is a factor that influences recovery or chronicity (Marshall et al., 2008; Zeeck et al., 2020). Additionally, shame is related to depressive symptoms (Orth et al., 2006; Guimón et al., 2007; Kim et al., 2011), and a recent meta-analysis shows that shame-related schemas are highly correlated with depression (Bishop et al., 2022). This evidence suggests that interventions focused on self-criticism and shame can be particularly effective for TRD.

Especially among CBIs, CFT is more expected to be effective because it was special developed with and for people with chronic mental problems (Gilbert and Procter, 2006; Gilbert and Simos, 2022). Compassion is viewed as a basic motive with processes for engaging with the causes and nature of a depression (e.g. develop emotion tolerance and empathy) and then develop the person’s courage and wisdom for a range helpful personable practices and actions (Gilbert, 2010). One central focus is self-criticism and shame (Gilbert, 2010; Gilbert and Simos, 2022). CFT hypothesizes that self-criticism and shame are influenced by a cold tone of self-talk toward the self and aims to transform it into a warm tone by developing compassion. To achieve this goal, some forms of psychoeducation are used in CFT to alleviate self-criticism and shame. Typical examples include the detailed psycho-eduction on the evolution-built nature of the motives and emotion process of the brain that can be very difficult to manage hence called the “tricky brain” which conveys a clear message that many of our difficult mental states like depression are linked to the activation of unwanted brain systems and states which is “not your fault” (Gilbert and Simos, 2022). In addition, patients are encouraged to practice a breathing technique—soothing breathing rhythm breathing—which helps bring them to a physiological state of grounding and slowing and enables a compassion focus for attention and reasoning. Accompanying these practices, patients work on techniques to directly develop compassionate images of themselves and others and practice acts of compassion (Gilbert and Simos, 2022). While addressing these processes, the therapist also fosters warmth and compassion in their relationship with the client. As an early report of CFT was for patient with chronic and severe mental health problems (Gilbert and Procter, 2006), CFT is a psychotherapy developed to help patients with chronic and severe problems like TRD.

As patients with TRD present severe and complex problems, exploring the feasibility of CFT for TRD can increase the number of available treatment options. Hence, this study evaluated the feasibility of group CFT for TRD in the Japanese population using an RCT design.

## Materials and methods

### Study design

This study was conducted as a prospective randomized controlled unmasked endpoint trial with two parallel groups and simple randomization at the individual level. Participants were randomly allocated to either a 12-week group CFT combined with usual care (UC; CFT group) or UC alone (UC group). Ethical approval was obtained from the Ethical Committee of the Safety and Health Organization, Chiba University (29-05), and the trial was registered at University hospital Medical Information Network (UMIN) (UMIN000028698).

### Participants and procedures

**FIGURE 1:**
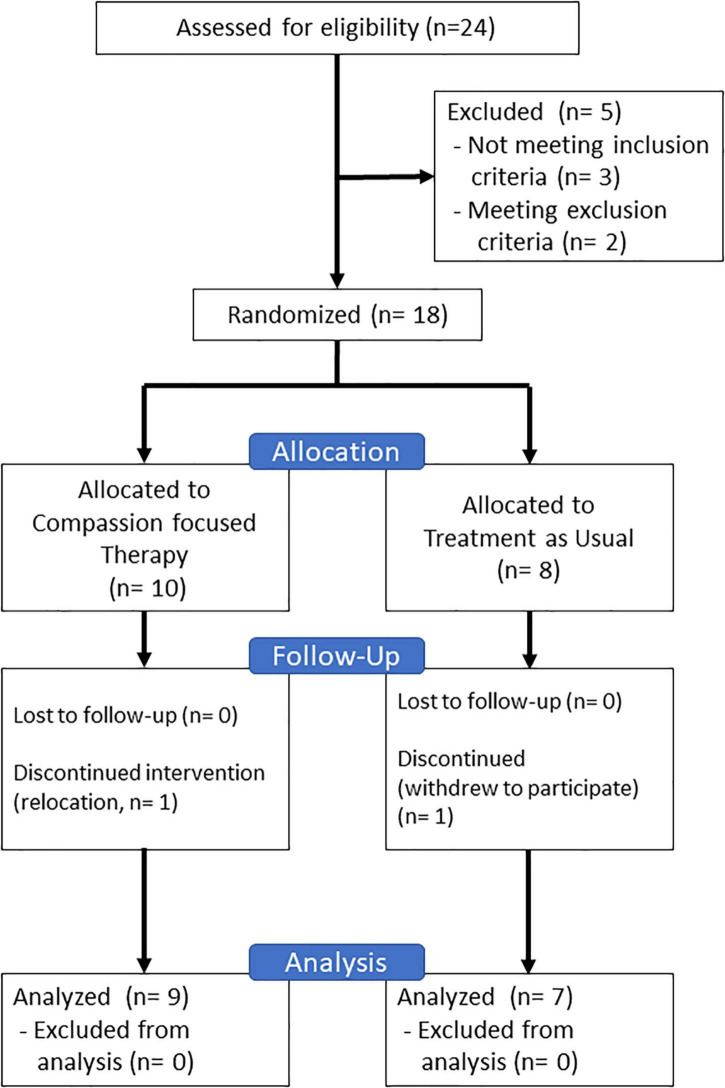
CONSORT diagram in this study.

The CONSORT diagram for the study is shown in Figure 1. Participants were recruited between July 2017 and September 2018 through posters and leaflets distributed at medical institutions in Chiba and Tokyo prefectures. Participants were asked to provide a referral letter from their psychiatrist with their application. After being fully apprised of the research, written informed consent was obtained from all the participants. They were then assessed based on the eligibility and exclusion criteria.

The inclusion criteria included: (a) primary diagnosis of major depressive disorder or dysthymia; (b) refractory to two Selective Serotonin Reuptake Inhibitor (SSRI) treatments; (c) while remaining at least moderately ill (Beck Depression Inventory-II (BDI-II) score ≧ 20; Kojima et al., 2002); (d) aged 18–60 years. We applied the criterion (a) above based on Diagnostic and Statistical Manual (DSM)-IV (American Psychiatric Association, 2000) because of the deficits of the structured interview tool based on DSM-5 in Japanese; we included major depressive disorder and dysthymia as diagnoses characterizing a depressive episode. We also applied criteria (b) and (c), based on the identification of TRD by Rush et al. (2006). Criterion (d) was adopted to avoid the need for parental consent. The exclusion criteria included: (1) psychosis; (2) active suicidality; (3) organic brain disorder; (4) substance abuse or dependence; (5) antisocial personality disorder; and/or (6) other severe mental/physical conditions. All exclusion criteria were set to ensure the protection and safety of the participants.

Participants were assessed using the Japanese version of the Mini-international Neuropsychiatric Interview (M.I.N.I.; Otsubo et al., 2005) to check their primary diagnosis (major depressive disorder or dysthymia) and comorbidity. BDI-II was then used to evaluate the participants’ symptoms and verify the inclusion criteria; whether patients were refractory to SSRI treatments was confirmed using the referral letters from their psychiatrists. Suicidal risk was evaluated based on the M.I.N.I. Even though an old version of the M.I.N.I. based on DSM-IV was used in this study, it was the latest available tool translated into Japanese.

After checking the eligibility and exclusion criteria, pre-intervention primary and secondary outcomes were measured. Post-intervention measurement was conducted after completing 12 weeks of sessions. The CFT group received a 12-week group CFT program in addition to UC. The CFT sessions were provided once a week, each session lasting 90 min. The UC group received care as usual during the 12 weeks. The number of participants ranged from four to six per group. A therapist and a co-therapist conducted the sessions. The therapist was a clinical psychologist with a Ph.D. in psychology who had trained at a three-day CFT workshop (KA). The co-therapist was an industrial counselor (YO). Both the therapist and co-therapist had completed a CBT training course at Chiba University (Kobori et al., 2014). Peer supervisions were conducted once a week by the therapist and co-therapist for quality control, and the fidelity checklist was confirmed. In addition, the therapist received supervision via a video meeting system from the other CFT therapists (PG or CI) once a month.

#### A 12-week group compassion-focused therapy program

The contents of the CFT program are shown in Table 1. The program materials were adapted from Judge et al. (2012) and translated into Japanese with adjustments based on Japanese culture. We also added psychoeducation about depressive symptoms to increase the participants’ understanding of their mental health problems.

**TABLE 1:** Contents of the group compassion-focused therapy program.

Session No.	Contents of Session
1	Psychoeducation of depression, tricky brain, and mindfulness
2	Three-circle model and soothing rhythm breathing
3	Psychoeducation of emotion, compassionate color or place
4	Psychoeducation for compassion and the compassionate self and memories
5 and 6	Images of compassionate self and others
7 and 8	Compassionate thinking
9	Case formulation for shame and self-criticism
10	Key fears and safety behaviors
11	Compassionate letter writing
12	Wrap-up and relapse prevention

In session 1, participants received psychoeducation about depression in terms of the tricky brain phenomenon, which is a popular and fundamental concept from the evolutionary psychology perspective (Gilbert and Simos, 2022). Mindfulness for sound exercise was also introduced and assigned to participants as homework. In session 2, participants were educated about the three-circle model, and the mechanisms of emotion and motivation were explained from a neuroscience perspective. Soothing rhythm breathing was introduced as homework. In session 3, participants learned about the functions of emotion and were assigned compassionate color and place image exercises as homework. Session 4 consisted of psychoeducation regarding compassion and the compassionate self for an understanding and developing a compassionate mind. Compassionate memory image exercises were introduced as homework. In sessions 5 and 6, participants were guided using image exercises pertaining to the compassionate self and others and asked to try the exercises at home.

In sessions 7 and 8, participants were taught to use compassionate images for switching from unhelpful thoughts to compassionate thinking using columns. Session 9 comprised a CFT case formulation to develop participant-specific formulations that reflected their vicious cycles. In session 10, participants were encouraged to replace their safety behaviors by following a case formulation to transform their cycles. Session 11 consisted of a compassionate letter writing exercise, wherein the participants were encouraged to write a letter addressed to themselves. Finally, in session 12, we summarized the program and devised plans to continue the participants’ efforts.

### Measures

#### Primary outcome

We used the BDI-II, which is the most popular self-reported questionnaire in clinical trials or meta-analyses, as the primary outcome (Beck et al., 1996; Kojima et al., 2002; Cuijpers et al., 2013). The BDI-II consists of 21 items, rated on a 4-point Likert scale ranging from 0 to 3. The score on the Japanese version of the BDI-II is classified as follows: 0–13, 14–19, 20–28, and 29–63, described as minimal, mild, moderate, and severe depression, respectively. The Japanese version of the BDI-II has been standardized and has demonstrated excellent reliability and validity. Cronbach’s α at pre-intervention was.73. Additionally, the BDI-II score was used as an inclusion criteria (participants who scored higher than 20 were included).

#### Secondary outcomes

We used the GRID-Hamilton Depression Rating Scale (GRID-HAMD), which has high reliability and validity, as the secondary outcome measure for depressive symptoms (Tabuse et al., 2007; Williams et al., 2008). The GRID-HAMD is a revised version of the Hamilton Depression Rating Scale, which is a widely-used structured interview that was developed by Hamilton (1960). The GRID-HAMD evaluates depressive symptoms on both intensity and frequency. The scale has two versions with 17 and 21 items, respectively. In this study, we used the 17-item version with scores ranging from 0 to 52 points. Cronbach’s α at pre-intervention was 0.58.

To assess the change in compassion, we used the Compassionate Engagement and Action Scales (CEAS). The CEAS was developed by Gilbert et al. (2017) and translated into Japanese (Asano et al., 2020); it includes three subscales of compassion (for others, from others, for self). The Japanese version has shown good reliability and validity. Each of the 3 subscales consists of 10 items rated on a Likert scale of 1 (*never*) to 10 (*always*). Cronbach’s α at pre-intervention were.94 (for others), 92 (from others),0.68 (for self).

To assess the resistance to developing compassion, we used the Fears of Compassion Scale (FCS; Gilbert et al., 2011). This reflects individuals’ problems in developing compassion, such as fear, resistance, or blocks that can be barriers to recovery. Kirby et al.’s (2019) meta-analysis showed that the fears of compassion are correlated with mental health difficulties. A cross-sectional study by Merritt and Purdon (2021) demonstrated that fears of compassion relate to ambivalence and the expectations regarding treatment. The FCS has been translated into Japanese and has demonstrated sufficient reliability and validity (Asano et al., 2017). It includes three subscales— fear of compassion for others, fear of compassion from others, and fear of compassion for self. The fear of compassion for others subscale consists of one factor with seven items. The fear of compassion from others contains two factors, namely, concern about compassion from others with four items, and avoidance of compassion from others with four items. The fear of compassion for self subscale comprises two factors: miserable with self-compassion, with five items, and demerit of self-compassion, with eight items. All the subscales are rated on a Likert scale of 0 (*do not agree at all*) to 4 (*completely agree*). Cronbach’s α at pre-intervention were 0.85 (for others), 69 (concern about compassion), 74 (avoidance of compassion from others), 80 (miserable with self-compassion), 65 (demerit of self-compassion).

To assess self-compassion (Neff, 2003), the Self-compassion Scale–Short Form (SCS–SF) was used. The SCS–SF is the short version of the Self-compassion Scale (SCS), and both SCS and SCS–SF have been translated into Japanese with high reliability and validity (Arimitsu, 2014; Arimitsu et al., 2016). SCS–SF and SCS include six factors. However, as most studies use the total score as a measure of self-compassion (MacBeth and Gumley, 2012; Zessin et al., 2015; Marsh et al., 2018; Liao et al., 2021), we also used the total score as the outcome measure. The SCS–SF consists of 12 items rated on a Likert scale of 1 (*almost never*) to 5 (*almost always*). Cronbach’s α at pre-intervention was 0.69.

We used a dropout rate and the Client Satisfaction Questionnaire (CSQ; Hisateru Tachimori, 1999) as indicators of the feasibility of the program. The CSQ consists of eight items rated on a Likert scale of 1 (*poor*) to 4 (*excellent*).

#### Evaluation of feasibility

Bowen et al. (2009) proposed eight areas of focus to discuss the feasibility of research on public health, four of which we examined in this study: acceptability, implementation, and limited efficacy testing. To evaluate acceptability, we used participants’ session attendance rates, dropout rates, and satisfaction levels as measured by CSQ. Implementation was evaluated according to the number of canceled, truncated, or postponed sessions. Therapist adherence was evaluated using a session checklist (part of the implementation of each session). Limited efficacy testing was evaluated based on differences in primary outcomes.

### Data analysis

All analyses were performed using R Statistical Software (v4.1.0; (R Core Team, 2021) and R Studio (v.2021.09.1 + 372 (Rstudio Team, 2021). To evaluate the change in outcomes, intent-to-treat linear mixed models were employed for all outcomes, assuming that the missing values occurred randomly, using lme4 package (v.1.1-27.1; (Bates et al., 2015)). The models included time, group, and time*group as the fixed effects and participant as a random effect. Standardized effect sizes for the difference from the baseline between conditions in linear mixed models were calculated using lme.dscore() function in EMAtools package (v.0.1.4 (Kleiman, 2021)).

We also performed Jacobson and Truax analysis to explore the clinically reliable indices (CRI) in the BDI-II score post intervention (Jacobson and Truax, 1991) using the R package JTRCI (Kruijt, 2021). The reliabilities were based on a previous BDI-II report confirming validity for a Japanese general sample (Kojima et al., 2002), and the mean score of 8.9 ± 6.5 was used as a norm (Kojima et al., 2002). The calculated cutoff point for recovery of c was 9.22; we therefore determined the cutoff point of recovery as less than 10 points on BDI-II.

## Results

### Participants’ characteristics

Twenty-four individuals consented to participate in the study. However, three of them did not meet the inclusion criteria, and two met the exclusion criteria. As a result, 18 participants were randomized, with 10 participants allocated to the CFT group and 8 to the UC group. A participant in the CFT group withdrew because of relocation. A participant in the UC group withdrew to participate while waiting and requested to have their data removed. Finally, the study comprised 17 participants, whose demographic and clinical characteristics are shown in Table 2.

**TABLE 2:** Participants’ characteristics.

Demographic variables		All (*n* = 17)	CFT (*n* = 10)	UC (*n* = 7)
Gender	Women, *n* (%)	15 (88)	8 (80)	6 (85)
Age (years)	Mean (*SD)*	39.88 (10.96)	39.8 (11.22)	40.0 (11.46)
	Median	42	42.5	38
	Range	28-56	24-56	26-55
Marital status, *n* (%)	Single	8 (47)	3 (30)	5 (71)
	Married	9 (53)	7 (70)	2 (29)
Educational background, N (%)	High school	6 (35)	4 (40)	2 (29)
	2 years vocational school	3 (18)	2 (20)	4 (57)
	≧ 3 years of college/university	7 (41)	3 (30)	1 (14)
	Graduate school	1 (6)	1 (10)	0 (0)
Duration of the current episode, years	Mean (*SD*)	7.89 (7.83)	4.57 (3.24)	12.63 (10.15)
	Median	5.67	3.08	9.91
	Range	1.33 − 28.58	1.33 − 10.0	2.0 − 28.58
Severity of treatment resistance (TRD score)	Mean (*SD*)	9.63 (1.02)	9.44 (1.13)	9.86 (0.90)
	Range	7 - 10	7 - 10	8 - 10
Primary diagnosis	Major depressive disorder	14 (82)	8 (80)	6 (86)
	Dysthymia	3 (18)	2 (20)	1 (14)
Comorbidity	Any psychiatric disorder	10 (59)	5 (50)	5 (71)
	Social anxiety disorder	6 (35)	4 (40)	4 (57)
	Generalized anxiety disorder	3 (18)	3 (30)	0 (0)
	Panic disorder	2 (12)	0 (0)	2 (29)
	Obsessive-compulsive disorder	2 (12)	1 (10)	2 (29)
	Post-Traumatic Stress Disorder	1 (6)	1 (10)	0 (0)
	Bulimia Nervosa	1 (6)	1 (10)	0 (0)

Based on the M.I.N.I. assessment, 14 participants were primarily diagnosed with major depressive disorder and the remaining 3 with dysthymia. Comorbidities were present as follows: six participants with social anxiety disorder, three with generalized anxiety disorder, two with panic disorder, two with obsessive-compulsive disorder, 1 with post-traumatic stress disorder, and one with bulimia nervosa. The TRD scores (Fekadu et al., 2009) were in the moderate range of 7 to 10 points, and the duration of the episode was over 4 years for all participants. Several other participants’ characteristics may have contributed to bias. For example, more participants in the CFT group were married than in the UC group (70% and 29%, respectively). Additionally, the duration of the current episode was longer among the UC group participants (UC = 12.63 years; CFT = 4.57).

### Changes in the primary outcome

The pre- and post-treatment assessments for both groups including interaction effects and between-group effects are presented in Table 3. The pre–post change for the BDI-II as the primary outcome was larger in the treatment group than in the control group post treatment, with a large between-group effect size of *d* = 2.30. The statistical results are provided in Table 3.

**TABLE 3:** Changes in outcomes.

		CFT		UC			
		
		pre	post	pre	post	Between-group differences	
		*n* = 10	*n* = 9	*n* = 7	*n* = 7	in pre–post change	
BDI-II	*M*	34.90	22.22	39.29	38.86	Δslope	11.62 [6.37, 16.88]
	*SD*	5.20	6.42	8.01	13.22	Effect size *d*	2.30
GRID-HAMD	*M*	16.20	10.00	16.43	15.50	Δslope	5.02 [−0.03, 10.07]
	*SD*	3.01	5.45	7.55	8.78	Effect size *d*	1.03
CEAS							
for self	*M*	53.20	67.89	47.00	42.43	Δslope	−19.05 [−37.04, −1.05]
	*SD*	12.44	12.47	14.90	20.18	Effect size *d*	−1.08
for others	*M*	73.78	82.22	55.86	58.83	Δslope	−8.15 [−22.17, 5.87]
	*SD*	14.36	9.05	18.28	26.10	Effect size *d*	−0.61
from others	*M*	55.78	59.78	53.86	57.14	Δslope	−2.17 [−18.95, 14.60]
	*SD*	24.05	24.62	17.16	18.22	Effect size *d*	−0.14
FCS							
for others	*M*	15.33	12.33	16.57	12.00	Δslope	−1.85 [−7.51, 3.83]
	*SD*	6.91	5.92	8.26	6.23	Effect size *d*	−0.34
Concern from others	*M*	10.30	9.78	10.57	9.00	Δslope	−1.10 [−4.08, 1.88]
	*SD*	2.63	3.73	4.47	4.86	Effect size *d*	−0.38
Avoidance from others	*M*	6.30	6.89	8.43	6.00	Δslope	−3.23 [−6.39, -0.07]
	*SD*	4.14	2.37	2.76	3.27	Effect size *d*	−1.06
Miserable with SC	*M*	10.10	7.78	13.57	13.86	Δslope	2.99 [−0.01, 5.99]
	*SD*	4.95	2.86	2.70	3.58	Effect size *d*	1.04
Demerit of SC	*M*	20.00	14.78	22.14	22.57	Δslope	5.73 [1.59, 9.86]
	*SD*	5.75	6.04	4.45	4.79	Effect size *d*	1.42
SCS–SF	*M*	33.90	36.22	35.71	35.14	Δslope	−2.57 [−5.62, 0.48]
	*SD*	2.77	2.44	3.50	3.72	Effect size *d*	−0.91

BDI-II, Beck Depression Inventory-II; GRID-HAMD, GRID-Hamilton Depression Rating Scale; CEAS, Compassionate Engagement and Action Scales; FCS, Fears of Compassion Scale; Concern from others, Concern about compassion from others; Avoidance from Others, Avoidance of compassion from others; Miserable with SC, Miserable with self-compassion; Demerit of SC, Demerit of self-compassion; SCS–SF, Self-compassion scale–short form.

The CRI indicated that three of nine participants recovered (33%), two improved (22%), two recovered but non-reliably (22%), and the condition of two remained unchanged (22%) in the CFT group. Only one patient non-reliably recovered and the other six patients’ conditions remained unchanged (17%) in the UC group.

### Changes in secondary outcomes

Similar to BDI-II, the pre–post change for the GRID-HAMD was larger in the CFT group than in the UC group post treatment, with a large between-group effect size of *d* = 1.03.

Regarding the CEAS scores, the post-treatment change on the compassion for self subscale was larger in the CFT group than in the UC group, with a large effect size of *d* = −1.08. There was a medium effect size of *d* = −0.61 on the compassion for others subscale.

With respect to the FCS, the post-treatment changes in the subscales “miserable with compassion for self” and “demerit of self-compassion” were larger in the CFT group than in the UC group, with large effect sizes of *d* = 1.04 and 1.42, respectively. Conversely, the post-treatment change on the avoidance of compassion from others subscale was larger in the UC group than in the CFT group, with a large effect size of *d* = −1.06. On the fears of compassion for others and concern about compassion from others subscales, there were medium effect sizes of *d* = −0.34 and −0.38.

The post-treatment change on SCS–SF (Neff, 2003) was larger in the CFT group than in the UC group, with a large effect size of *d* = −0.91.

### Evaluation of feasibility

On acceptability, attendance rates were from 75 to 100%, 2 participants were absent once and 1 participant was absent twice and 1 participant was absent three times. The remaining 4 participants attended all sessions. The rate of intervention completion was 90% (dropout rate was 10%). The mean Client Satisfaction Questionnaire score was 28.17 (*SD* = 2.29), and all responses were higher than 3 (*good*). On implementation, all sessions were conducted as planned even if some of the participants were absent, so no sessions were canceled, truncated, or postponed sessions. All components of session check list were conducted as planned. Limited efficacy testing was as described in 3.2 section.

## Discussion

In this study, we verified the feasibility of group CFT for TRD using an RCT design. Building on the scarce literature on the effectiveness of CBIs for chronic depression or TRD (Graser et al., 2016), our results illustrate the possibility of using CFT to treat patients with TRD. Furthermore, as studies investigating the effectiveness of CBIs for the Japanese population are very limited (Arimitsu, 2016; Asano and Shimizu, 2018; Asano, 2019), our study also demonstrates the possibility of using CFT or CBIs in the Japanese context. A cross-cultural study indicated that the Japanese population has the highest self-criticism among 13 countries (Halamová et al., 2018); hence, it is beneficial to verify the feasibility of CFT, which focuses on self-criticism (Gilbert, 2010), for Japan.

### Participants’ characteristics in this study

In terms of the participants’ demographic data, the percentage of men and the mean duration of major depression were low compared to those found by a previous study (Jaffe et al., 2019); however, the education level was higher, whereas age and marital status were almost equivalent on average. The low proportion of men participants may have been because of the available time frame for participation, subject to gender roles in Japanese society, and the differences in resistance to the group format across genders. The reason for the high percentage of college graduates is unknown but may be related to the program being provided at a university campus.

Duration of the current episode was considerably greater for UC group. Although episode length relates to severity and comorbidity (Melartin et al., 2004), measured severity and comorbidity in this study were not seemed to be differed between groups. It is not easy to identify which factor affected to biased duration of the current episode, there is a possibility that a few people with longer duration were assigned to the UC group. In any case, the result of this study should be carefully interpreted with considering for this bias.

Approximately 60% of the participants had psychiatric comorbidities. This was in line with the findings of previous studies, which showed that more than half of the TRD patients presented anxiety disorders or other psychiatric problems (Casher et al., 2012; Kubitz et al., 2013; Huang et al., 2020; Fabbri et al., 2021).

### Changes in the primary outcome

A recent meta-analytic review by Cuijpers et al. (2020) showed that the effect size *g* of CBT ranged from 0.50 to 0.69 compared to the care-as-usual group; for problem-solving therapy, which showed the largest effect size, *g* ranged from 0.42 to 2.05. Regarding group CBT, an old meta-analysis by McDermut et al. (2001) reported the effect size *d* as 1.03 (range: −0.07 to 2.30, 48.2%). Based on studies of CBIs, Kirby et al. (2017) revealed that the effect size *d* of CBI ranged from 0.45 to 0.82. Wilson et al. (2019) estimated that the effect size *g* of self-compassion-related interventions ranged from 0.23 to 0.57. Considering these reports, the effect size on BDI-II in this study using a therapy developed for and with patients was large (*d* = 2.30), suggesting that this program can be a new treatment choice for patients with TRD. However, unchanged symptoms in the UC group should be considered. A recent review reported that 12.5% of patients with depression remit within 12 weeks even when untreated (Mekonen et al., 2022). In a similar study among the Japanese population, the score for depressive symptoms reduced in the treatment as usual group (Nakagawa et al., 2017). In contrast, the BDI-II score in the UC group in this study remained at the same level during the research period. This may be related to the inclusion criteria we used, such as patients with TRD being at least moderately ill prior to the study.

The CRI results showed that five of nine (56%) participants with TRD responded (recovered or improved) in the CFT group, whereas only one of seven (14%) responded in the UC group. Cuijpers et al. (2021) have pointed out that 41% of patients with psychiatric diagnosis respond to psychotherapy. Another CBT trial by Wiles et al. (2013) for TRD reported that 46% of patients recovered. A meta-analysis of group CBT by McDermut et al. (2001) indicated that 48.2% of patients showed improvement. Although differences in calculation exist, the CRI of this program can be deemed sufficient.

### Changes in secondary outcomes

With regard to GRID-HAMD scores, the effect size was large and showed sufficient reduction in depressive symptoms. The difference in effect sizes between GRID-HAMD and BDI-II may have been caused by discrepancies in the two measurements. The BDI-II is associated with the psychological symptoms of depression and is more sensitive when patients are older (over 50 years old), with higher neuroticism or atypical depression, compared to GRID-HAMD (Enns et al., 2000). Therefore, CFT can be expected to be more effective in addressing psychological symptoms of depression and atypical depression that may lead to chronic depression.

### Compassion-related outcomes

First, we found that in the CFT group, compassion for self increased; two factors of fears of compassion for self (demerits of self-compassion and miserable with self-compassion) decreased greatly. Cuppage et al. (2018) showed that decreased fears of compassion for self are correlated with changes in psychopathology. Hence, we may assume that depressive symptoms reduced via increasing compassion for self and decreasing fears of compassion for self.

Second, compassion for others increased more in the CFT group than in the UC group. Increased compassion for others may be associated with the intervention delivered in the CFT program. Guided imagery, in which one directed compassion toward others, along with other skills included in the CFT program may have stimulated participants’ compassionate attitudes toward others. We also found that fears of compassion for others decreased in both groups, but the decrease was higher in the UC group. Therefore, it cannot be concluded that fears of compassion for others decreased because of the intervention.

Third, no great difference was observed between the groups in terms of the change in compassion from others, but fears of compassion from others showed a greater reduction in the UC group. These results were also unexpected. However, compassion from others may be more affected by interpersonal relationships beyond therapy.

Finally, SCS–SF scores showed a greater increase in the CFT group, which can be considered as an indicator of participants’ recovery. Previous research has shown that increased self-compassion predicts improved symptoms (Galili-Weinstock et al., 2018). Therefore, our program is likely to have resulted in improving depressive symptoms via self-compassion.

### Evaluation of feasibility

CFT is designed to reduce patients’ shame and prevent dropouts (Lucre and Corten, 2013). However, there is not enough evidence to conclude that the dropout rate is low for this therapy. The program in this study had a low dropout rate (10%), a high attendance rate (from 75% to 100%), and high CSQ scores. Although further RCTs with larger sample sizes are needed to verify the CFT dropout rate, our program showed sufficient acceptability. Moreover, as all sessions were conducted as planned, the implementation was also acceptable.

### Limitations and future directions

This study is subject to some limitations. First, the sample size was small, most of the participants were women, and bias may have affected the between-group “duration of the current episode” and “comorbidity.” Hence, larger and balanced samples are needed to verify the program’s effectiveness. Second, sampling biases should also be considered: our study was primarily conducted in Japan’s metropolitan areas of Chiba and Tokyo, and the range of application was limited to patients with TRD who remained at least moderately ill. Thus, program feasibility should be examined with participants from other regions and cultural areas and with various depressive episode histories. Third, the results of this study cannot determine the mechanism of CFT. Variables that affect treatment should be measured during the treatment period using larger samples, particularly because reduction in fears of compassion for others and from others were observed in both CFT and UC groups. Steps should be taken to examine the role of relevant variables to further refine treatment, especially using longitudinal surveys. Fourth, there were no indicators to evaluate group dynamics, which is considered to be an important factor in group psychotherapy. Qualitative data from observation and objective measurements are also needed to optimize the effectiveness of the intervention.

Notwithstanding these limitations, the group CFT program showed adequate feasibility, and preliminary efficacy for TRD. Psychological treatments for TRD are limited; hence, CFT can be a new treatment option. The program follows a group format, which is more cost effective and resolves the shortage of therapists. Further research is warranted to improve the robustness of these findings.

## Data availability statement

The raw data supporting the conclusions of this article will be made available by the authors, without undue reservation.

## Ethics statement

The studies involving human participants were reviewed and approved by Ethical Committee of the Safety and Health Organization, Chiba University. The patients/participants provided their written informed consent to participate in this study.

## Author contributions

KA initiated and wrote a protocol for ethical approval of the study and manuscript, and worked as the therapist. YO worked as the co-therapist. TSe, AM, and AI conducted structured interviews (GRID-HAMD) for the independent evaluation. MT conducted the analysis and wrote the analysis and results sections of the manuscript with KA. TO and MS assessed the inclusion criteria from the perspective of participants’ safety. KA, TSh, and ES managed recruitment, and PG supervised the intervention. CI developed the original program of intervention and advised KA to implement the program for the Japanese population. All authors contributed to the article and approved the submitted version.

## Abbreviations

TRD: treatment-resistant depression
CFT: compassion focused therapy
UC: usual care
CBT: cognitive behavioral therapy
CBI: compassion-based interventions
BDI-II: beck depression inventory-II
GRID-HAMD: GRID-Hamilton depression rating scale
CEAS: compassionate engagement and action scales
FCS: fears of compassion scale
SCS–SF: self-compassion scale–short-form.

## References

[B1] Al-Harbi K. S. (2012). Treatment-resistant depression: therapeutic trends, challenges, and future directions. *Patient Prefer Adherence* 6 369–388. 10.2147/PPA.S29716 22654508 PMC3363299

[B2] American Psychiatric Association (2000). *Diagnostic and Statistical Manual of Mental Disorders*, 4th ed., text revision Edn. Washington, DC: American Psychiatric Association.

[B3] American Psychological Association (2019). *Clinical Practice Guideline for the Treatment of Depression Across Three age Cohorts.* Washington, DC: American Psychological Association.

[B4] Arimitsu K. (2014). Development and validation of the Japanese version of the self-compassion scale. *Shinrigaku Kenkyu* 85 50–59. 10.4992/jjpsy.85.50 24804430

[B5] Arimitsu K. (2016). The effects of a program to enhance self-compassion in Japanese individuals: A randomized controlled pilot study. *J. Posit. Psychol.* 11 559–571. 10.1080/17439760.2016.1152593

[B6] Arimitsu K. Aoki Y. Furukita M. Tada A. Togashi R. (2016). Construction and validation of a short form of the Japanese version of the Self-Compassion Scale. *Komazawa Annu. Rep. Psychol.* 18 1–9.

[B7] Asano K. (2019). Emotion processing and the role of compassion in psychotherapy from the perspective of multiple selves and the compassionate self. *Case Rep. Psychiatry* 2019:7214752. 10.1155/2019/7214752 30723566 PMC6339704

[B8] Asano K. Shimizu E. (2018). A case report of compassion focused therapy (CFT) for a Japanese patient with recurrent depressive disorder: the importance of layered processes in CFT. *Case Rep. Psychiatry* 2018:4165434. 10.1155/2018/4165434 30416837 PMC6207871

[B9] Asano K. Kotera Y. Tsuchiya M. Ishimura I. Lin S. Matsumoto Y. (2020). The development of the Japanese version of the compassionate engagement and action scales. *PLoS One.* 15:e0230875. 10.1371/journal.pone.0230875 32236112 PMC7112184

[B10] Asano K. Tsuchiya M. Ishimura I. Lin S. Matsumoto Y. Miyata H. (2017). The development of fears of compassion scale Japanese version. *PLoS One.* 12:e0185574. 10.1371/journal.pone.0185574 29023461 PMC5638239

[B11] Bates D. Mächler M. Bolker B. Walker S. (2015). Fitting linear mixed-effects models using lme4. *J. Stat. Softw.* 67 1–48. 10.18637/jss.v067.i01

[B12] Beck A. T. Steer R. A. Ball R. Ranieri W. (1996). Comparison of beck depression inventories -IA and -II in psychiatric outpatients. *J. Pers. Assess.* 67 588–597. 8991972 10.1207/s15327752jpa6703_13

[B13] Bishop A. Younan R. Low J. Pilkington P. D. (2022). Early maladaptive schemas and depression in adulthood: A systematic review and meta-analysis. *Clin. Psychol. Psychother.* 29 111–130. 34131990 10.1002/cpp.2630

[B14] Bowen D. J. Kreuter M. Spring B. Cofta-Woerpel L. Linnan L. Weiner D. (2009). How we design feasibility studies. *Am. J. Prev. Med.* 36 452–457. 19362699 10.1016/j.amepre.2009.02.002 PMC2859314

[B15] Brown L. Houston E. E. Amonoo H. L. Bryant C. (2021). Is self-compassion associated with sleep quality? A meta-analysis. *Mindfulness* 12 82–91. 10.1007/s12671-020-01498-0

[B16] Casher M. I. Gih D. Agarwala P. (2012). Confounding factors in treatment-resistant depression (Part 2). Comorbidities and treatment resistance. *Psychiatr. Times* 29 43–43.

[B17] Craig C. Hiskey S. Spector A. (2020). Compassion focused therapy: a systematic review of its effectiveness and acceptability in clinical populations. *Exp. Rev. Neurother.* 20 385–400. 10.1080/14737175.2020.1746184 32196399

[B18] Cuijpers P. Berking M. Andersson G. Quigley L. Kleiboer A. Dobson K. S. (2013). A meta-analysis of cognitive-behavioural therapy for adult depression, alone and in comparison with other treatments. *Can. J. Psychiatry* 58 376–385. 10.1177/070674371305800702 23870719

[B19] Cuijpers P. Karyotaki E. Ciharova M. Miguel C. Noma H. Furukawa T. A. (2021). The effects of psychotherapies for depression on response, remission, reliable change, and deterioration: a meta-analysis. *Acta Psychiatr. Scand.* 144 288–299. 10.1111/acps.13335 34107050 PMC8457213

[B20] Cuijpers P. Karyotaki E. de Wit L. Ebert D. D. (2020). The effects of fifteen evidence-supported therapies for adult depression: A meta-analytic review. *Psychother. Res.* 30 279–293. 10.1080/10503307.2019.1649732 31394976

[B21] Cuppage J. Baird K. Gibson J. Booth R. Hevey D. (2018). Compassion focused therapy: exploring the effectiveness with a transdiagnostic group and potential processes of change. *Br. J. Clin. Psychol.* 57 240–254. 10.1111/bjc.12162 29044607

[B22] EMEA (2009). CHMP. Concept Paper on the Need for Revision of Note for Guidance on Clinical Investigation of Medicinal Products in the Treatment of Depression with Regard to Treatment Resistant Depression. Available online at: https://www.ema.europa.eu/en/documents/scientific-guideline/concept-paper-need-revision-guideline-clinical-investigation-medicinal-products-treatment-depression_en.pdf (accessed July 10, 2022).

[B23] Enns M. W. Larsen D. K. Cox B. J. (2000). Discrepancies between self and observer ratings of depression. The relationship to demographic, clinical and personality variables. *J. Affect. Disord.* 60 33–41. 10.1016/s0165-0327(99)00156-1 10940445

[B24] Fabbri C. Hagenaars S. P. John C. Williams A. T. Shrine N. Moles L. (2021). Genetic and clinical characteristics of treatment-resistant depression using primary care records in two UK cohorts. *Mol. Psychiatry* 26 3363–3373. 10.1038/s41380-021-01062-9 33753889 PMC8505242

[B25] Fekadu A. Wooderson S. C. Markopoulou K. Cleare A. J. (2009). The Maudsley Staging Method for treatment-resistant depression: prediction of longer-term outcome and persistence of symptoms. *J. Clin. Psychiatry* 70 952–957. 19457299 10.4088/JCP.08m04728

[B26] Ferrari M. Ciarrochi J. Yap K. Sahdra B. Hayes S. C. (2022). Embracing the complexity of our inner worlds: Understanding the dynamics of self-compassion and self-criticism. *Mindfulness* 13, 1652–1661. 10.1007/s12671-022-01897-5

[B27] Galili-Weinstock L. Chen R. Atzil-Slonim D. Bar-Kalifa E. Peri T. Rafaeli E. (2018). The association between self-compassion and treatment outcomes: session-level and treatment-level effects. *J. Clin. Psychol.* 74 849–866. 10.1002/jclp.22569 29251782

[B28] Gilbert P. (2010). An introduction to compassion focused therapy in cognitive behavior therapy. *Int. J. Cogn. Ther.* 3 97–112. 10.1521/ijct.2010.3.2.97

[B29] Gilbert P. Procter S. (2006). Compassionate mind training for people with high shame and self-criticism: Overview and pilot study of a group therapy approach. *Clin. Psychol. Psychother.* 13 353–379.

[B30] Gilbert P. Simos G. (eds) (2022). *Compassion Focused Therapy: Clinical Practice and Applications.* Oxfordshire, UK: Routledge.

[B31] Gilbert P. Catarino F. Duarte C. Matos M. Kolts R. Stubbs J. (2017). The development of compassionate engagement and action scales for self and others. *J. Compassionate Health Care* 4:4. 10.1186/s40639-017-0033-3

[B32] Gilbert P. McEwan K. Matos M. Rivis A. (2011). Fears of compassion: development of three self-report measures. *Psychol. Psychother.* 84 239–255. 10.1348/147608310X526511 22903867

[B33] Graser J. Höfling V. Weβlau C. Mendes A. Stangier U. (2016). Effects of a 12-week mindfulness, compassion, and loving kindness program on chronic depression: a pilot within-subjects wait-list controlled trial. *J. Cogn. Psychother.* 30 35–49. 10.1891/0889-8391.30.1.35 32755904

[B34] Guimón J. Las Hayas C. Guillén V. Boyra A. González-Pinto A. (2007). Shame, sensitivity to punishment and psychiatric disorders. *Eur. J. Psychiatry* 21 124–133.

[B35] Halamová J. Kanovský M. Gilbert P. Troop N. A. Zuroff D. C. Hermanto N. (2018). The factor structure of the forms of self-criticising/attacking & self-reassuring scale in thirteen distinct populations. *J. Psychopathol. Behav. Assess.* 40 736–751. 10.1007/s10862-018-9686-2 30459486 PMC6223807

[B36] Hamilton M. (1960). The Hamilton Depression Scale—accelerator or break on antidepressant drug discovery. *Psychiatry* 23 56–62. 10.1136/jnnp-2013-306984 24443712

[B37] Hisateru Tachimori H. I. (1999). Reliability and validity of the Japanese version on client satisfaction questionnaire. *Clin. Psychiatry* 41 711–717.

[B38] Huang S. S. Chen H. H. Wang J. Chen W. J. Chen H. C. Kuo P. H. (2020). Investigation of early and lifetime clinical features and comorbidities for the risk of developing treatment-resistant depression in a 13-year nationwide cohort study. *BMC Psychiatry* 20:541. 10.1186/s12888-020-02935-z 33203427 PMC7672820

[B39] Ijaz S. Davies P. Williams C. J. Kessler D. Lewis G. Wiles N. (2018). Psychological therapies for treatment-resistant depression in adults. *Cochrane Database Syst. Rev.* 5:CD010558. 10.1002/14651858.CD010558.pub2 29761488 PMC6494651

[B40] Jacobson N. S. Truax P. (1991). Clinical significance: a statistical approach to defining meaningful change in psychotherapy research. *J. Consult. Clin. Psychol.* 59 12–19. 10.1037//0022-006x.59.1.12 2002127

[B41] Jaffe D. H. Rive B. Denee T. R. (2019). The humanistic and economic burden of treatment-resistant depression in Europe: a cross-sectional study. *BMC Psychiatry* 19:247. 10.1186/s12888-019-2222-4 31391065 PMC6686569

[B42] James S. L. Abate D. Abate K. H. Abay S. M. Abbafati C. Abbasi N. (2018). Global, regional, and national incidence, prevalence, and years lived with disability for 354 diseases and injuries for 195 countries and territories, 1990–2017: a systematic analysis for the Global Burden of Disease Study 2017. *Lancet* 392 1789–1858. 10.1016/S0140-6736(18)32279-7 30496104 PMC6227754

[B43] Judge L. Cleghorn A. McEwan K. Gilbert P. (2012). An exploration of group-based compassion focused therapy for a heterogeneous range of clients presenting to a community mental health team. *Int. J. Cogn. Ther.* 5 420–429. 10.1521/ijct.2012.5.4.420

[B44] Kim S. Thibodeau R. Jorgensen R. S. (2011). Shame, guilt, and depressive symptoms: a meta-analytic review. *Psychol. Bull.* 137:68. 10.1037/a0021466 21219057

[B45] Kirby J. N. Day J. Sagar V. (2019). The “Flow” of compassion: A meta-analysis of the fears of compassion scales and psychological functioning. *Clin. Psychol. Rev.* 70 26–39. 10.1016/j.cpr.2019.03.001 30884253

[B46] Kirby J. N. Tellegen C. L. Steindl S. R. (2017). A meta-analysis of compassion-based interventions: current state of knowledge and future directions. *Behav. Ther.* 48 778–792. 10.1016/j.beth.2017.06.003 29029675

[B47] Kleiman E. (2021). *EMAtools: Data Management Tools for Real-Time Monitoring/Ecological Momentary Assessment Data. R package version 0.1.4.* Available online at: https://CRAN.R-project.org/package=EMAtools (accessed July 10, 2022).

[B48] Kobori O. Nakazato M. Yoshinaga N. Shiraishi T. Takaoka K. Nakagawa A. (2014). Transporting cognitive behavioral therapy (CBT) and the Improving Access to Psychological Therapies (IAPT) project to Japan: preliminary observations and service evaluation in Chiba. *J. Ment. Health Train. Educ. Pract.* 9 155–166. 10.1108/JMHTEP-10-2013-0033

[B49] Kojima M. Furukawa T. A. Takahashi H. Kawai M. Nagaya T. Tokudome S. (2002). Cross-cultural validation of the Beck Depression Inventory-II in Japan. *Psychiatry Res.* 110 291–299. 10.1016/s0165-1781(02)00106-3 12127479

[B50] Kruijt A.-W. (2021). *JTRCI: Obtain and Plot Jacobson-Truax and Reliable Change Indices. R Package Version 0.1.0.* Available online at: https://github.com/AWKruijt/JT-RCI (accessed July 10, 2022).

[B51] Kubitz N. Mehra M. Potluri R. C. Garg N. Cossrow N. (2013). Characterization of treatment resistant depression episodes in a cohort of patients from a US commercial claims database. *PLoS One* 8:e76882. 10.1371/journal.pone.0076882 24204694 PMC3799999

[B52] Liao K. Y.-H. Stead G. B. Liao C.-Y. (2021). A meta-analysis of the relation between self-compassion and self-efficacy. *Mindfulness* 12 1878–1891. 10.1007/s12671-021-01626-4

[B53] Lucre K. M. Corten N. (2013). An exploration of group compassion-focused therapy for personality disorder. *Psychol. Psychother.* 86 387–400. 10.1111/j.2044-8341.2012.02068.x 24217864

[B54] MacBeth A. Gumley A. (2012). Exploring compassion: a meta-analysis of the association between self-compassion and psychopathology. *Clin. Psychol. Rev.* 32 545–552. 10.1016/j.cpr.2012.06.003 22796446

[B55] Marsh I. C. Chan S. W. Y. MacBeth A. (2018). Self-compassion and psychological distress in adolescents-a meta-analysis. *Mindfulness* 9 1011–1027. 10.1007/s12671-017-0850-7 30100930 PMC6061226

[B56] Marshall M. B. Zuroff D. C. McBride C. Bagby R. M. (2008). Self-criticism predicts differential response to treatment for major depression. *J. Clin. Psychol.* 64 231–244. 18302208 10.1002/jclp.20438

[B57] McDermut W. Miller I. W. Brown R. A. (2001). *The Efficacy of Group Psychotherapy for Depression: a Meta-Analysis and Review of the Empirical Research.* York, EN: Centre for Reviews and Dissemination.

[B58] Mekonen T. Ford S. Chan G. C. K. Hides L. Connor J. P. Leung J. (2022). What is the short-term remission rate for people with untreated depression? A systematic review and meta-analysis. *J. Affect. Disord.* 296 17–25. 10.1016/j.jad.2021.09.046 34583099

[B59] Melartin T. K. Rytsälä H. J. Leskelä U. S. Lestelä-Mielonen P. S. Sokero T. P. Isometsä E. T. (2004). Severity and comorbidity predict episode duration and recurrence of DSM-IV major depressive disorder. *J. Clin. Psychiatry* 65 19397. 10.4088/jcp.v65n0612 15291658

[B60] Merritt O. A. Purdon C. (2021). Fear of compassion is associated with treatment ambivalence and negative expectations for treatment in people with anxiety. *Br. J. Clin. Psychol.* 60 546–555. 10.1111/bjc.12313 34117792

[B61] Muris P. Otgaar H. (2022). Deconstructing self-compassion: How the continued use of the total score of the self-compassion scale hinders studying a protective construct within the context of psychopathology and stress. *Mindfulness* 13, 1403–1409. 10.1007/s12671-022-01898-4 35578653 PMC9095813

[B62] Nakagawa A. Mitsuda D. Sado M. Abe T. Fujisawa D. Kikuchi T. (2017). Effectiveness of supplementary cognitive-behavioral therapy for pharmacotherapy-resistant depression: A randomized controlled trial. *J. Clin. Psychiatry.* 78 1126–1135. 10.4088/JCP.15m10511 28252882

[B63] National Collaborating Centre for Mental Health (2011). *Depression: the Treatment and Management of Depression in Adults (Updated Edition).* Leicester UK: British Psychological Society. 22132433

[B64] National Institute for Health and Care Excellence (2009). *Depression in Adults: Recognition and Management. Treatments for Mild-to-Moderate Depression. Clinical Guideline [CG90].* Available online at https://www.nice.org.uk/guidance/cg90 (accessed July 10, 2022).

[B65] Neff K. D. (2003). The development and validation of a scale to measure self-compassion. *Self Identity* 2 223–250. 10.1080/15298860309027 26979311

[B66] Nishi D. Ishikawa H. Kawakami N. (2019). Prevalence of mental disorders and mental health service use in Japan. *Psychiatry Clin. Neurosci.* 73 458–465. 10.1111/pcn.12894 31141260

[B67] Orth U. Berking M. Burkhardt S. (2006). Self-conscious emotions and depression: Rumination explains why shame but not guilt is maladaptive. *Pers. Soc. Psychol. Bull.* 32 1608–1619. 17122174 10.1177/0146167206292958

[B68] Otsubo T. Tanaka K. Koda R. Shinoda J. Sano N. Tanaka S. (2005). Reliability and validity of Japanese version of the Mini-International Neuropsychiatric Interview. *Psychiatry Clin. Neurosci.* 59 517–526. 10.1111/j.1440-1819.2005.01408.x 16194252

[B69] Phillips W. J. Hine D. W. (2021). Self-compassion, physical health, and health behaviour: a meta-analysis. *Health Psychol. Rev.* 15 113–139. 10.1080/17437199.2019.1705872 31842689

[B70] R Core Team (2021). *R: A Language and Environment for Statistical Computing.* Vienna: R Foundation for Statistical Computing.

[B71] Rstudio Team (2021). *RStudio: Integrated Development Environment for R.* Boston, MA: RStudio.

[B72] Rush A. J. Trivedi M. H. Wisniewski S. R. Nierenberg A. A. Stewart J. W. Warden D. (2006). Acute and longer-term outcomes in depressed outpatients requiring one or several treatment steps: a STAR*D report. *Am. J. Psychiatr.* 163 1905–1917. 10.1176/ajp.2006.163.11.1905 17074942

[B73] Tabuse H. Kalali A. Azuma H. Ozaki N. Iwata N. Naitoh H. (2007). The new GRID Hamilton Rating Scale for Depression demonstrates excellent inter-rater reliability for inexperienced and experienced raters before and after training. *Psychiatry Res.* 153 61–67. 17445908 10.1016/j.psychres.2006.07.004

[B74] Thase M. E. (2011). Treatment-resistant depression: prevalence, risk factors, and treatment strategies. *J. Clin. Psychiatry* 72:e18. 10.4088/JCP.8133tx4c 21658343

[B75] Thomas L. Kessler D. Campbell J. Morrison J. Peters T. J. Williams C. (2013). Prevalence of treatment-resistant depression in primary care: cross-sectional data. *Br. J. Gen. Pract.* 63 e852–e858. 10.3399/bjgp13X675430 24351501 PMC3839394

[B76] Wiles N. Thomas L. Abel A. Ridgway N. Turner N. Campbell J. (2013). Cognitive behavioural therapy as an adjunct to pharmacotherapy for primary care based patients with treatment resistant depression: results of the CoBalT randomised controlled trial. *Lancet* 381 375–384. 10.1016/S0140-6736(12)61552-9 23219570

[B77] Williams J. B. W. Kobak K. A. Bech P. Engelhardt N. Evans K. Lipsitz J. (2008). The GRID-HAMD: standardization of the Hamilton Depression Rating Scale. *Int. Clin. Psychopharmacol.* 23 120–129. 10.1097/YIC.0b013e3282f948f5 18408526

[B78] Wilson A. C. Mackintosh K. Power K. Chan S. W. Y. (2019). Effectiveness of self-compassion related therapies: a systematic review and meta-analysis. *Mindfulness* 10 979–995.

[B79] Wong M. Y. C. Chung P.-K. Leung K.-M. (2021). The relationship between physical activity and self-compassion: a systematic review and meta-analysis. *Mindfulness* 12 547–563.

[B80] Zeeck A. Von Wietersheim J. Weiss H. Hermann S. Endorf K. Lau I. (2020). Self-criticism and personality functioning predict patterns of symptom change in major depressive disorder. *Front. Psychiatry* 11:147. 10.3389/fpsyt.2020.00147 32226398 PMC7081790

[B81] Zessin U. Dickhäuser O. Garbade S. (2015). The relationship between self-compassion and well-being: A meta-analysis. *Appl. Psychol. Health Well. Being* 7 340–364. 10.1111/aphw.12051 26311196

